# Musquitoes

**Published:** 1870-10

**Authors:** 


### MUSQUITOES.

It is said that no musquito will alight on a spot where coal oil has been applied. Try it.

